# Factors Associated with Normal-Weight Abdominal Obesity Phenotype in a Representative Sample of the Peruvian Population: A 4-Year Pooled Cross-Sectional Study

**DOI:** 10.3390/jcm12103482

**Published:** 2023-05-16

**Authors:** Jamee Guerra Valencia, Lorena Saavedra-Garcia, Víctor Juan Vera-Ponce, Rubén Espinoza-Rojas, Noel C. Barengo

**Affiliations:** 1 Facultad de Ciencias de la Salud, Universidad Privada del Norte, Lima 15314, Peru; 2 Facultad de Ciencias de la Salud, Universidad San Ignacio de Loyola, Lima 15024, Peru; 3 Instituto de Investigaciones en Ciencias Biomédicas (INICIB), Universidad Ricardo Palma, Lima 15039, Peruruben.espinoza@urp.edu.pe (R.E.-R.); 4 Facultad de Psicología, Universidad Tecnológica del Perú, Lima 15046, Peru; 5 Herbert Wertheim College of Medicine, Florida International University, Miami, FL 33199, USA; nbarengo@fiu.edu; 6 Faculty of Medicine, Riga Stradins University, LV-1007 Riga, Latvia

**Keywords:** obesity, abdominal obesity, normal body mass index, risk factors, Latin America

## Abstract

To examine factors associated with abdominal obesity among normal-weight individuals from the Demographic and Health Survey of Peru (2018–2021). Cross-sectional analytical study. The outcome variable was abdominal obesity defined according to JIS criteria. Crude (cPR) and adjusted prevalence ratios (aPR) were estimated for the association between sociodemographic and health-related variables and abdominal obesity using the GLM Poisson distribution with robust variance estimates. A total of 32,109 subjects were included. The prevalence of abdominal obesity was 26.7%. The multivariate analysis showed a statistically significant association between abdominal obesity and female sex (aPR: 11.16; 95% CI 10.43–11.94); categorized age 35 to 59 (aPR: 1.71; 95% CI 1.65–1.78); 60 to 69 (aPR: 1.91; 95% CI 1.81–2.02); and 70 or older(aPR: 1.99; 95% CI 1.87–2.10); survey year 2019 (aPR: 1.22; 95% CI 1.15–1.28); 2020 (aPR: 1.17; 95% CI 1.11–1.24); and 2021 (aPR: 1.12; 95% CI 1.06–1.18); living in Andean region (aPR: 0.91; 95% CI 0.86–0.95); wealth index poor (aPR: 1.26; 95% CI 1.18–1.35); middle (aPR: 1.17; 95% CI 1.08–1.26); rich (aPR: 1.26; 95% CI 1.17–1.36); and richest (aPR: 1.25; 95% CI 1.16–1.36); depressive symptoms (aPR: 0.95; 95% CI 0.92–0.98); history of hypertension (aPR: 1.08; 95% CI 1.03–1.13), type 2 diabetes (aPR: 1.13; 95% CI 1.07–1.20); and fruit intake 3 or more servings/day (aPR: 0.92; 95% CI 0.89–0.96). Female sex, older ages, and low and high income levels increased the prevalence ratio for abdominal obesity, while depressive symptoms, living in the Andean region, and fruit intake of 3 or more servings/day decreased it.

## 1. Introduction

Cardiovascular diseases are the major cause of premature mortality, leading to close to 18 million deaths per year [1]. Excessive body adiposity has been shown to be an important risk factor for various metabolic and cardiovascular diseases [2,3,4,5]. Although body mass index (BMI) is commonly used to assess excess adiposity in both clinical and research settings [6,7], BMI fails to differentiate between peripheral and central adipose tissue distribution. Studies have revealed that the accumulation of abdominal fat, in particular visceral adipose tissue (VAT), is a stronger predictor of metabolic and dyslipidemia disorders than subcutaneous adipose tissue [3,4,8]. There has been an increase in abdominal obesity and annual growth rates since the 1990s [9,10]. This trend increases the public health burden, as evidenced by the high costs associated with it worldwide [11,12,13,14]. For instance, the total public health expenditure for cardiometabolic diseases in China amounted to USD 30,350.8 million, of which 28.7% was attributable to abdominal obesity, exceeding even the expenditure associated with general obesity (12.7%) [15]. Similarly, healthcare spending on type 2 diabetes mellitus (DM2) and hypertension (HTA) in older adults in Brazil was found to be higher among those with abdominal obesity compared to their counterparts with normal WC [16]. 

Numerous studies have demonstrated that WC is associated with a greater risk of developing DM2, HTA, cardiovascular diseases, cardiovascular mortality, and total mortality, even among individuals with a normal BMI [17,18,19,20]. Current scientific evidence further suggests that the normal-weight abdominal obesity phenotype may be present in people with a normal BMI but an increased WC [2,19]. There is a lack of evidence of this phenotype in Latin America; however, some studies show a wide range between countries. For instance, a study in Chile found 12.4% in men and 16.4% in women; in contrast, a Peruvian study found a prevalence of 51.7% [21,22]. Despite the high prevalence of this phenotype [19], it often remains undetected as BMI is the common measure of body composition in clinical practice, and individuals with a normal BMI are typically not prioritized for prevention programs. 

Although numerous genetic and environmental factors can influence abdominal obesity [23], including sex, age, and ethnicity [24,25,26,27], there is a paucity of research focused on the factors associated with abdominal obesity in individuals with a normal BMI. Furthermore, given the rapid growth of central obesity, particularly in Latin America [10,28], and the considerable burden it poses to public health [16], it is important to have more scientific evidence in the region. 

The objective of this study was to examine the factors associated with the abdominal obesity phenotype in a representative sample of the Peruvian population with a normal BMI from 2018 to 2021.

## 2. Materials and Methods

### 2.1. Study Design

This cross-sectional analytical study used secondary data from the Demographic and Health Survey (ENDES, by its acronym in Spanish). For this study, data collected from 2018, 2019, 2020, and 2021 were analyzed. The STROBE (Strengthening the Reporting of Observational Studies in Epidemiology) guidelines were followed by the present study [29].

### 2.2. Population and Sample

**Figure 1:**
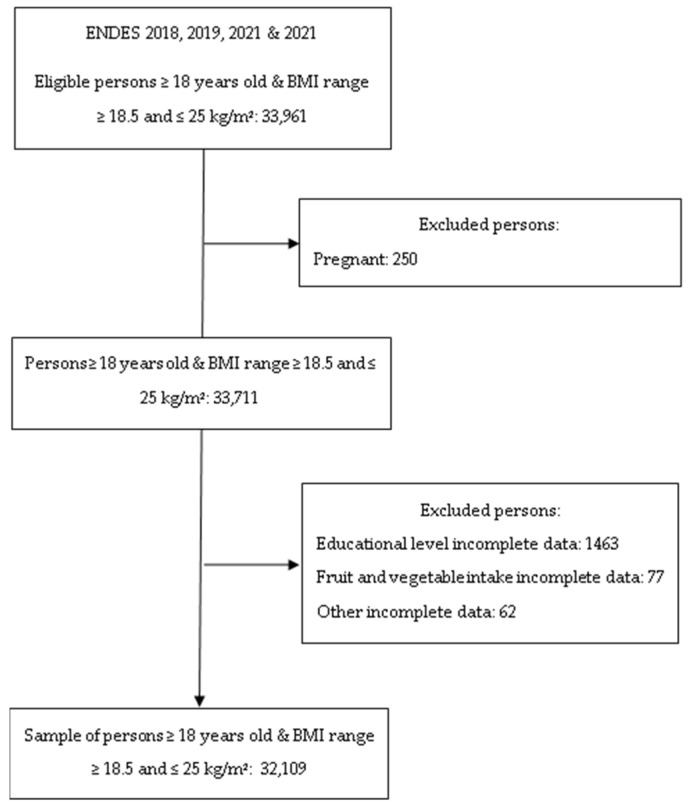
Flow diagram of the participant selection included in the study.

The ENDES surveys are carried out by the National Institute of Statistics and Informatics and use balanced, two-stage, stratified, and independent probability sampling at the departmental level and in urban and rural areas. Comprehensive information on the methods used in this study has been described elsewhere [30]. The study population was defined considering the following inclusion criteria: (1) adults of both sexes aged 18 years or older; (2) subjects with a body mass index ≥18.5 and ≤24.9 kg/m^2^; and (3) subjects with complete information on the variables of interest for the current research. The exclusion criteria included pregnant women and those with incomplete information on the key variables (Figure 1).

### 2.3. Variables and Measurements

The main outcome variable for this study was the normal-weight abdominal obesity phenotype, which was defined as the presence of a BMI between 18.5 and 24.9 kg/m^2^ and a WC ≥ 94 cm and 80 cm for males and females [31], respectively. BMI was calculated using the weight/height^2^ formula. 

Height was measured with a mobile, multipurpose wooden stadiometer with a precision of 1 mm and following the technical specifications provided by the National Food and Nutrition Center (CENAN, by its acronym in Spanish). Body weight was measured with a SECA-878 brand scale with a precision of 50 g, while waist circumference was measured using a Lufkin brand retractable metal tape with a resolution of 0.1. The anthropometric techniques recommended by the WHO were followed to measure weight, height, and waist circumference, with the latter being measured as the mean distance between the last costal margin and the upper edge of the iliac crest, as previously reported in the ENDES anthropometric manuals [32]. 

The independent variables of the study to be evaluated were sex (male vs. female); age (18–35, 36–59, 60–69, and 70 years or older); year of survey (2018, 2019, 2020, and 2021); natural region (Metropolitan Lima, rest of the coast, Andean, and Amazon); area of residence (urban vs. rural); educational level (no education, primary, secondary, and higher); wealth index (poorest, poor, middle, rich, and richest); daily smoking (yes vs. no); self-reported alcohol intake in the previous 12 months (yes vs. no); the presence of depressive symptoms in the 14 days prior to the survey as determined by a score of five or more in the Patient Health Questionnarie-9 screening test (yes vs. no); self-reported DM2 (yes vs. no); HTA (yes vs. no) defined as an average blood pressure (two readings) ≥ 140 mmHg systolic blood pressure and/or diastolic blood pressure ≥ 90 mmHg or previously diagnosed by a physician; self-reported fruit intake of 3 or more servings per day (yes vs. no); and self-reported vegetable intake of 2 or more servings per day (yes vs. no). 

### 2.4. Statistical Analysis

STATA version 17 statistical software was used. The prevalence of the normal-weight abdominal obesity phenotype was estimated, and Chi-square tests were used for each possible factor associated with the normal-weight abdominal obesity phenotype. Finally, crude (cPR) and adjusted (aPR) prevalence ratios were calculated using generalized linear models with robust variance estimation, assuming a Poisson distribution with logarithmic link functions. All analyses were performed considering that they were complex samples. An alpha of 0.05 was chosen for statistical significance.

### 2.5. Ethics Considerations

The National Institute of Statistics and Informatics (INEI) collects and maintains the data from the ENDES surveys. All data were fully anonymized prior to being made available on the internet at (http://iinei.inei.gob.pe/microdatos/ accessed on 10 February 2023). Since ethical approval was obtained from the institution that commissioned, funded, and managed the overall DHS Program (the ICF Institutional Review Board (IRB), https://dhsprogram.com/Methodology/Protecting-the-Privacy-of-DHS-Survey-Respondents.cfm accessed on 10 February 2023), further ethical approval was not required. Informed consent was obtained from the participants before the survey.

## 3. Results

A total of 32,109 subjects were included in the study. The female sex represented 40.7% of the study population. More than half (51.5%) were between 18 and 35 years old; a third lived in metropolitan Lima; and most lived in urban settings (74%). Most of the individuals denied smoking (98.2%) and alcohol intake (87.8%), while only 7.6% reported a vegetable intake of 2 or more servings per day. Depressive symptoms, hypertension, and type 2 diabetes had a prevalence of 20.4%, 6.4%, and 2.9%, respectively, while abdominal obesity was prevalent in 26.7% of the study population (Table 1).

The bivariate analysis showed a statistically significant association between all the evaluated factors and the normal-weight abdominal obesity phenotype (Table 2).

After adjustment for the independent variables of the study, the multivariate analysis (Table 3) found a statistically significant association between the normal-weight abdominal obesity phenotype and female sex (PRa: 11.16; 95% CI 10.43–11.94); categorized age 35 to 59 (PRa: 1.71; 95% CI 1.65–1.78); 60 to 69 (PRa: 1.91; 95% CI 1.81–2.02); and 70 or older (PRa: 1.99; 95% CI 1.87–2.10); survey year collection 2019 (PRa: 1.22; 95% CI 1.15–1.28); 2020 (PRa: 1.17; 95% CI 1.11–1.24); and 2021 (PRa: 1.12; 95% CI 1.06–1.18); living in the Andean region (PRa: 0.91; 95% CI 0.86–0.95); wealth index poor (PRa: 1.26; 95% CI 1.18–1.35); middle (PRa: 1.17; 95% CI 1.08–1.26); rich (PRa: 1.26; 95% CI 1.17–1.36); and richest (PRa: 1.25; 95% CI 1.16–1.36); depressive symptoms (PRa: 0.95; 95% CI 0.92–0.98); history of hypertension (PRa: 1.08; 95% CI 1.03–1.13), type 2 diabetes (PRa: 1.13; 95% CI 1.07–1.20); and fruit intake in a dose of 3 or more servings per day (PRa: 0.92; 95% CI 0.89–0.96).

Moreover, upon stratifying the data by sex, an increasing adjusted prevalence ratio (aPR) was observed among men as they advanced in age. In contrast, women displayed a statistically significant association with normal-weight abdominal obesity that persisted irrespective of age. Additionally, the relationship between wealth index and normal-weight abdominal obesity varied between men and women. Among men, there was an increase in the prevalence ratio for abdominal obesity among normal BMI subjects with an increasing wealth index. In contrast, among women, the aPR remained relatively consistent across different wealth index levels (Table A1).

## 4. Discussion

### 4.1. Main Findings

Our data revealed that female sex and older age, type 2 diabetes mellitus, and hypertension were positively associated with abdominal obesity in normal-weight individuals. On the other hand, the presence of depressive symptoms, living in the Andean region, and fruit intake at a dose of three or more servings per day decreased the probability of a normal-weight abdominal obese phenotype. We also found that the wealth-rich index and the year of the national survey collection were associated with the studied phenotype.

### 4.2. Comparison with Other Studies

The prevalence of abdominal obesity identified in this study was consistent with a prior investigation conducted in Peru [22], albeit nearly three times higher than that reported in studies of normal BMI individuals in the United States [18,19]. The high prevalence found in our study can be partially attributed to ethnic differences, with previous research indicating that Latin Americans have a greater propensity for visceral adiposity accumulation than other ethnic groups [27,33]. These findings highlight the significance of abdominal obesity among normal BMI individuals, particularly considering the accelerating increase in abdominal obesity within the general population of the Latin American region, with South America being particularly impacted [10]. 

The female sex was strongly associated with a normal-weight abdominal obesity phenotype when compared with its male counterparts. Although there is little scientific information on this phenotype, there is evidence that abdominal obesity affects women more severely than men in the general population [9,10,18,22,24,34]. While the role of sexual dimorphism in adiposity distribution has been previously acknowledged [23], and women typically tend to deposit more adipose tissue in the gluteofemoral region than in the abdominal area, it has also been reported that patterns of adipose distribution in women exhibit greater variability among ethnic groups than in men [23]. In line with this, a study that assessed genomic ancestry contribution to abdominal obesity showed that Peruvian and Mexican populations presented the highest Native-American ancestry among other Latin American countries, and this was in turn positively associated with an abdominal fat distribution more severely present in female sex [33]. Furthermore, the same study showed that while Peruvian and Mexican women exhibited an android body shape, women from Chile tended more toward a gynoid body shape [33]. Various loci have been identified in the Latin American population related to different anthropometric central adiposity measures, with variations within Latinos [35]. This may reflect the significant differences among countries in the Latin American region, for which caution is suggested when extrapolating the present findings to other countries in the region. 

Age was significantly associated with the normal-weight abdominal obesity phenotype, and its magnitude was further increased with increasing ages. This was not an unexpected finding since it is well recognized that body fat distribution is affected by aging with a characterized pattern for reduced appendicular fat, increased trunk fat, mainly abdominal fat, and fat infiltration in organs such as the liver and skeletal muscle [36]. In support of our findings, different studies have previously reported a central obesity increase in risk as the population ages [10,18,33,37,38,39,40]. Furthermore, an increase in abdominal fat in older age occurs along with the accumulation of fat infiltration into non-fat tissues, which further increases the metabolic risk [36]. In support of this, a cohort study with a mean follow-up of 6.5 years found that among older people with normal BMI, both coronary artery disease and mortality risk increased by 1.65 times in the highest WC tertile compared with the lowest one [41]. 

Stratified analysis by sex showed a greater adjusted prevalence ratio (aPR) among men as they aged and increased their wealth index, while this pattern did not occur in women. Socio-cultural as well as biopsychological factors may potentially explain these findings. For instance, it has been reported that self-perceptions of BMI and WC are commonly underestimated [42,43,44], and this tendency may vary between men and women [44]. Notably, in a cohort of middle-aged EPIC-Oxford participants, men were found to consistently underestimate their WC to a greater extent than women, and this pattern became more pronounced as actual WC increased [44]. In addition, the accumulation of adipose tissue in the visceral region is known to be associated with the aging process [36]. Hence, normal-weight men may not fully realize the potential dangers of incremental waist circumference and may underestimate their WC. Furthermore, local cultural factors in Peru may contribute to this underestimation [43], and a greater wealth index may lead to “technological sedentarism”, which entails increased access to motorized transport and office work that promote less physical activity [45]. Taken together, these socio-cultural and biopsychological factors may help explain the differences found in the present study.

Type 2 diabetes mellitus, hypertension, and depressive symptoms were also associated with abdominal obesity among normal-weight people. These findings are consistent with several studies [4,17,18,19,46,47]. Notably, in a prospective study with a 7-year follow-up period, an increase in waist circumference among individuals with normal BMI was found to increase the risk of developing DM2 by sixfold [17]. Furthermore, the association between hypertension and abdominal obesity among individuals with normal BMI has been documented in cross-sectional studies conducted in populations such as that of the United States. In fact, the risk was nearly two-fold higher than that observed among individuals without abdominal obesity and a normal BMI [18]. Several studies have demonstrated an association between an increase in waist circumference and a higher risk of cardiovascular mortality, even in cases where BMI falls within the normal range [4,19,46,47]. The underlying mechanisms that explain these findings are related to visceral fat deposition patterns, which may trigger insulin resistance, systemic inflammation, and oxidative stress that eventually leads to cardiometabolic disturbances [4]. Additionally, it has been well established that there exists a bidirectional relationship between abdominal obesity and depressive symptoms, leading to an elevated risk of each condition [45,46,47,48,49,50]. Recently, a prospective study revealed that higher depression levels at baseline increased abdominal obesity risk during the 7-year follow-up [49]. However, it is worth noting that this study did not stratify abdominal obesity development risk by BMI categories, which could have potentially underestimated depressive symptoms’ effects on weight loss. In support of this, another prospective study found that higher depression scores at baseline were a strong predictor of both weight gain and weight loss after a 3-year follow-up period [51].

Living in the Andean region was associated with a lower prevalence of the normal-weight abdominal obesity phenotype. These findings may be explained by the altitude effect on general and central adiposity. Several studies that include cross-sectional and experimental designs have found an inverse association between acute and chronic altitude exposure and general and abdominal obesity prevalence when compared to low altitude [34,39,52,53,54,55,56,57]. For instance, in the Tibetan population aged 30–70 years old, a reduction in BMI, WC, and the waist-to-height ratio was found as altitude residence increased [54]. In the same way, evidence from the USA population consistently showed that high-altitude dwellers displayed a lower obesity/central obesity [52,56,57] and DM2 [57] risk when compared to lowlanders. Studies from Peru, a country belonging to the Andean region of South America with roughly 25% of the population living at or above 3000 m above sea level [58], have found that irrespective of the criterion used to define abdominal obesity, high altitude is negatively associated with central obesity [34,39], a pattern that was mainly shown in men rather than women [34]. The biological mechanism underlying adiposity reduction in high altitude is still not completely understood; however, recent evidence suggests that this reduction seen in hypoxic environments seems largely due to decreased energy intake secondary to a yet unexplained decrease in appetite mechanisms, which may or may not be accompanied by an increase in energy expenditure resulting from an increase in resting metabolic rate and physical activity [34,52,54,56,59]. In line with the above, physical activity has consistently been greater among highlanders when compared to lowlanders [34,52,54,56], as has weight loss when low-intensity physical exercise in normobaric hypoxia was compared to the same intensity exercise performed in sham normobaric hypoxia in obese patients [53]. 

Fruit intake in a dose at or above 3 servings a day was significantly associated with a reduction of roughly 9% in the normal-weight abdominal obesity phenotype. However, 2 or more servings per day of vegetable consumption did not show a significant association. These specific fruit and vegetable doses were assessed in the present study on the rational basis of previous existing evidence pointing out a protective effect of these doses against obesity [60] and cardiometabolic diseases [61,62]. Abdominal obesity risk reduction by fruit intake among normal BMI individuals is not an unexpected finding, as we have previously reported an inverse association between intake for each fruit serving and WC in about 0.4 cm of reduction, which increased to 0.6 cm of reduction when we assessed a dose of 3 or more servings per day [60]. In line with the above, a prospective study with 5.5 years of follow-up found that the change in waist circumference for a given BMI was −0.04 cm/year for fruit intake [63]. The same study showed that when replacing 100 kcal of high-fat and high-sugar foods with about 1 fruit serving size, there was a significant reduction in WC adjusted for BMI that fluctuated between −0.08 and −0.05 cm/year [63]. These findings support the idea that fruit intake plays a protective role in central adiposity accretion regardless of BMI status. On the other hand, vegetable intake was not associated with a normal-weight abdominal obesity phenotype, a finding that does not comport with previous evidence [63,64,65]. This may be partially explained by the fact that vegetable intake in the present study was self-reported through a question that specifically inquired about vegetable salad consumption. Because many culinary differences exist in vegetable salad elaboration in Peru, which may even include starchy vegetables as the main ingredient (i.e., potatoes, sweet potatoes, corn) and high-caloric salad dressings [66], a significant association with vegetables may have been neglected.

The wealth index was positively associated with abdominal obesity among normal BMI individuals. Our findings showed that both low and high-wealth indexes displayed a risk for abdominal fat accretion. Previous studies have shown that the wealth index is related to central obesity [10,57,67,68,69]. However, the associated pattern may differ between countries’ income levels. For instance, in low-middle-income countries, such as Peru, both low and high socio-economic status may show a risk for central obesity [38,69,70], while in high-income countries, a high wealth index is mainly associated with abdominal obesity [9,57]. This may be explained by the nutritional transition that in developing countries started later but experienced rapid changes due to the reduction of costs of fast foods and ultra-processed foods, allowing access to these foods to a less wealthy population, who find these products more affordable and convenient than healthy diets [28]. 

The data from the 2019 national survey showed a greater prevalence of the normal-weight abdominal obesity phenotype compared to the other survey years. These findings match the per capita gross domestic product (GDP) in Peru in the 2018–2021 period [71], with 2019 being the highest in this timeframe. This may explain the greater prevalence ratio of central obesity among normal BMI individuals during 2019, as increases in the GDP per capita in low- and middle-income countries have been found to predict an increase in general adiposity [72]. On the contrary, in 2020, GDP per capita showed the lowest level in the past 5 years. This, along with the epidemic COVID-19 and the associated confinement, may have increased sedentary behavior and decreased compliance with healthy dietary habits (i.e., a decrease in fruit and vegetable intake and an increase in sweets and dessert consumption, among others) [67,73], which in turn may have increased abdominal obesity prevalence. Furthermore, the release of sanitary restrictions imposed during the COVID-19 pandemic displayed in Peru in the year 2021 may explain the decreased prevalence ratio for the normal-weight abdominal obesity phenotype when compared to 2020.

### 4.3. Implications of the Study

Our findings revealed several important aspects regarding abdominal obesity in the Peruvian population. First, abdominal obesity is highly prevalent in normal-weight individuals, as almost 27% of normal-weight people showed an elevated WC. Considering that for each 1 cm increase in WC, a 2% increased risk of cardiovascular disease has been reported [74] and that abdominal obesity is rapidly growing among South American countries [10,28], our findings on the normal-weight abdominal obesity phenotype are of interest to public health policy development. Therefore, routine WC measurement should be encouraged both at the clinical and population levels, as the reduction in WC, regardless of BMI status, is a cost-effective strategy that can be achieved by routine, moderate-intensity exercise and/or dietary interventions [75]. Moreover, considering that individuals in the low socioeconomic Peruvian population who are overweight or obese according to BMI are unaware of the severity of their weight status [43], this approach might help to appropriately target this vulnerable population. 

Second, our research showed a higher prevalence ratio for abdominal obesity in normal-weight individuals that more severely affects women and the older Peruvian population. Because not only the prevalence of general obesity but abdominal obesity within normal BMI more severely affects women than men, targeting women in health interventions should be highly considered, as women’s nutritional status has been shown to be associated with their offspring’s nutritional status [76]. Furthermore, pre-gestational abdominal obesity has been shown to be associated with adverse pregnancy outcomes [77]. In addition, early intervention in women can also have a positive impact on their own health, reducing the risk of non-communicable diseases and improving the overall quality of life. A higher prevalence ratio of abdominal obesity with aging in normal-weight individuals as early as the third decade of life was shown in our study. This finding highlights the importance of public health policies focusing on healthy aging as abdominal obesity prevalence is increasing [10]. 

Third, it is important to consider that the normal-weight abdominal obesity phenotype, which was associated with DM2 and HTA, is not a benign phenotype. A pattern of abdominal obesity [2] has been shown to increase the health care burden, as previously reported by others [15,16], highlighting the need for encouraging WC measurement even in normal BMI individuals. Fourth, while socioeconomic inequalities among abdominal obesity in the general population have been documented [67], our study suggests that central obesity is currently affecting both extremes of income distribution at an almost similar magnitude among individuals with a normal BMI. Therefore, interventions aimed at reducing the cardiometabolic burden should be tailored to the needs of each income group rather than adopting a “one size fits all” approach. Finally, living in the Andean region and having an adequate fruit intake may serve as protective factors against abdominal obesity. These findings are of gravitate relevance as Peru is a country with about 25% of the population living at high altitudes [58], and learning from the factors that mediate the protective effects may help to better design intervention programs. Additionally, as fruit intake is well recognized to be part of healthy diets and may help lower food and nutrition insecurity [78,79], enhancing nutrition education and food accessibility through national policies should be considered. 

### 4.4. Study Limitations

Naturally, our study has some limitations. First, the study’s cross-sectional nature prevents the establishment of a causal relationship between the outcomes obtained. Second, although in the analysis multiple sociodemographic and personal variables were assessed, physical activity, energy intake and expenditure, and other lifestyle variables that may be associated (i.e., sleep quality) were not considered as this data was not available in the national survey. Third, fruit and vegetable intake was assessed via self-report; however, the intake assessment was carried out with open questions about the frequency per week and portions per day of consumption of fruits and vegetable salads. This was based on the proposal of the World Health Organization, the STEPwise approach to noncommunicable disease risk-factor surveillance [80]. Fourth, it should be noted that the diagnosis of DM2 was based on self-reporting rather than clinical and laboratory criteria, which may have led to an underestimation of DM2 prevalence and the strength of its association with the normal-weight abdominal obesity phenotype, given that DM2 is often underdiagnosed.

## 5. Conclusions

The study shows that one-third of the normal-weight adult Peruvian population presents with abdominal obesity. Some socioeconomic factors were positively associated, and others were the opposite. The evidence should prompt policymakers to focus health promotion and prevention initiatives on factors related to this phenotype and specific segments of the population. Additionally, it is necessary to raise awareness about this common phenotype among Peruvians due to its high prevalence and its implication in the development of non-communicable diseases.

## Figures and Tables

**Table 1:** Characteristics of the study sample.

Characteristics	*n* (%Weighted)	95% CI
**Sex**		
Male	19,041 (59.3)	58.4–60.2
Female	13,068 (40.7)	39.8–41.6
**Age (years)**		
18 to 35	16,534 (51.5)	50.5–52.4
35 to 59	9982 (31.1)	30.2–31.9
60 to 69	2722 (8.5)	8.0–9.0
≥70	2871 (8.9)	8.4–9.6
**Survey year**		
2018	6411 (20.0)	19.2–20.7
2019	10,045 (31.3)	30.4–32.2
2020	6526 (20.3)	19.4–21.3
2021	9128 (28.4)	27.3–29.6
**Natural region**		
Metropolitan Lima	10,896 (33.9)	32.6–35.3
Rest of the coast	6938 (21.6)	20.6–22.6
Andean	9693 (30.2)	29.0–31.4
Amazon	4582 (14.3)	13.5–15.1
**Educational level**		
No education	101 (0.3)	0.2–0.4
Primary	7511 (23.4)	22.6–24.2
Secondary	13,738 (42.8)	41.9–43.7
Higher	10,759 (33.5)	32.5–34.5
**Wealth index**		
Poorest	8283 (25.8)	24.9–26.7
Poor	6951 (21.6)	20.8–22.5
Middle	5943 (18.5)	17.8–19.3
Rich	5617 (17.5)	16.6–18.4
Richest	5316 (16.6)	15.6–17.6
**Residence area**		
Urban	23,754 (74.0)	73.0–74.9
Rural	8355 (26.0)	25.1–27.0
**Daily smoking**		
No	31,529 (98.2)	97.9–98.4
Yes	580 (1.8)	1.6–2.1
**Alcohol intake**		
No	28,184 (87.8)	87.1–88.5
Yes	3913 (12.2)	11.5–12.9
**Depressive symptoms**		
No	25,554 (79.6)	78.9–80.3
Yes	6555 (20.4)	19.7–21.1
**Hypertension**		
No	30,042 (93.6)	93.1–94.1
Yes	2046 (6.4)	5.9–6.9
**Type 2 diabetes**		
No	31,178 (97.1)	96.7–97.5
Yes	921 (2.9)	2.5–3.3
**Fruit intake ≥3 servings/day**		
No	25,399 (79.1)	78.3–79.9
Yes	6710 (20.9)	20.1–21.7
**Vegetable intake ≥2 servings/day**		
No	29,673 (92.4)	91.9–92.9
Yes	2436 (7.6)	7.1–8.1
**Abdominal obesity**		
No	23,537 (73.3)	72.5–74.1
Yes	8572 (26.7)	25.9–27.5

**Table 2:** Bivariate characteristics of the factors associated with the normal-weight abdominal obesity phenotype.

Characteristics	Normal-Weight Abdominal Obese		
	No	Yes	*p* *
	*n* (%)	*n* (%)	
**Sex**			
Male	18,122 (95.2)	920 (4.8)	
Female	5416 (41.4)	7652 (58.6)	<0.001
**Age (years)**			
18 to 35	13,241 (80.1)	3293 (19.9)	<0.001
35 to 59	6761 (67.7)	3221 (32.3)	
60 to 69	1783 (65.5)	938 (34.5)	
≥70	1751 (61.0)	1120 (39.0)	
**Survey year**			
2018	5498 (85.8)	913 (14.2)	<0.001
2019	6955 (69.2)	3089 (30.8)	
2020	4564 (69.9)	1962 (30.1)	
2021	6520 (71.4)	2607 (28.6)	
**Natural region**			
Metropolitan Lima	7445 (68.3)	3451 (31.7)	<0.001
Rest of the coast	4914 (70.8)	2024 (29.2)	
Andean	7647 (78.9)	2047 (21.1)	
Amazon	3532 (77.1)	1051 (22.9)	
**Educational level**			
No education	65 (64.7)	36 (35.3)	<0.001
Primary	5259 (70.0)	2252 (30.0)	
Secondary	10,647 (77.5)	3092 (22.5)	
Higher	7566 (70.3)	3192 (29.7)	
**Wealth index**			
Poorest	6760 (81.6)	1523 (18.4)	<0.001
Poor	5300 (76.3)	1651 (23.7)	
Middle	4392 (73.9)	1551 (26.1)	
Rich	3841 (68.4)	1775 (31.6)	
Richest	3244 (61.0)	2071 (39.0)	
**Residence area**			
Urban	16,771 (70.6)	6983 (29.4)	<0.001
Rural	6766 (81.0)	1588 (19.0)	
**Daily smoking**			
No	23,044 (73.1)	8485 (26.9)	<0.001
Yes	494 (85.1)	86 (14.9)	
**Alcohol intake**			
No	20,232 (71.8)	7952 (28.2)	<0.001
Yes	3295 (84.2)	618 (15.8)	
**Depressive symptoms**			
No	19,003 (74.4)	6551 (25.6)	<0.001
Yes	4534 (69.2)	2021 (30.8)	
**Hypertension**			
No	22,358 (74.4)	7684 (25.6)	<0.001
Yes	1161 (56.7)	885 (43.3)	
**Type 2 diabetes**			
No	23,050 (73.9)	8128 (26.1)	<0.001
Yes	481 (52.2)	440 (47.8)	
**Fruit intake ≥3 per day**			
No	18,307 (72.1)	7092 (27.9)	<0.001
Yes	5230 (77.9)	1480 (22.1)	
**Vegetable intake ≥2 per day**			
No	21,823 (73.5)	7849 (26.5)	0.001
Yes	1714 (70.4)	722 (29.6)	

* Analysis performed with the Chi-square test for independence. Significant *p*-value < 0.05.

**Table 3:** Simple and adjusted multivariate regression analysis of factors associated with the normal-weight abdominal obesity phenotype.

Characteristics	Crude Model PR (CI 95%)	Adjusted Model ** PR (CI 95%)
**Sex**		
Male	Ref.	Ref.
Female	**11.92 (11.16–12.73)**	**11.16 (10.43–11.94)**
**Age (years)**		
18 to 35	Ref.	Ref.
35 to 59	**1.72 (1.64–1.79)**	**1.71 (1.65–1.78)**
60 to 69	**1.81 (1.71–1.93)**	**1.91 (1.81–2.02)**
70 and older	**2.07 (1.96–2.19)**	**1.99 (1.87–2.10)**
**Survey year**		
2018	Ref.	Ref.
2019	**2.14 (2.00–2.30)**	**1.22 (1.15–1.28)**
2020	**2.11 (1.97–2.27)**	**1.17 (1.11–1.24)**
2021	**1.98 (1.85–2.13)**	**1.12 (1.06–1.18)**
**Natural region**		
Metropolitan Lima	Ref.	Ref.
Rest of the coast	**0.92 (0.88–0.96)**	1.03 (0.99–1.07)
Andean	**0.67 (0.63–0.7)**	**0.91 (0.86–0.95)**
Amazon	**0.68 (0.63–0.72)**	0.96 (0.91–1.02)
**Educational level**		
No education	Ref.	Ref.
Primary	0.85 (0.65–1.11)	1.23 (0.98–1.54)
Secondary	**0.62 (0.47–0.81)**	1.18 (0.94–1.48)
Higher	0.82 (0.63–1.07)	1.21 (0.96–1.52)
**Wealth index**		
Poorest	Ref.	Ref.
Poor	**1.33 (1.24–1.42)**	**1.26 (1.18–1.35)**
Middle	**1.46 (1.37–1.57)**	**1.17 (1.08–1.26)**
Rich	**1.79 (1.68–1.9)**	**1.26 (1.17–1.36)**
Richest	**2.21 (2.08–2.35)**	**1.25 (1.16–1.36)**
**Residence area**		
Urban	Ref.	Ref.
Rural	**0.63 (0.60–0.67)**	0.97 (0.91–1.03)
**Daily smoking**		
No	Ref.	Ref.
Yes	**0.57 (0.46–0.69)**	1.01 (0.87–1.18)
**Alcohol intake**		
No	Ref.	Ref.
Yes	**0.57 (0.53–0.61)**	1.01 (0.95–1.07)
**Depressive symptoms**		
No	Ref.	Ref.
Yes	**1.20 (1.15–1.26)**	**0.95 (0.92–0.98)**
**Hypertension**		
No	Ref.	Ref.
Yes	**1.71 (1.62–1.8)**	**1.08 (1.03–1.13)**
**Type 2 diabetes**		
No	Ref.	Ref.
Yes	**1.82 (1.7–1.96)**	**1.13 (1.07–1.20)**
**Fruit intake ≥3 per day**		
No	Ref.	Ref.
Yes	**0.79 (0.75–0.83)**	**0.92 (0.89–0.96)**
**Vegetable intake ≥2 per day**		
No	Ref.	Ref.
Yes	**1.11 (1.04–1.19)**	1.05 (1.00–1.10)

** Adjusted for sex, age, survey year collection, natural region, educational level, wealth index, residence area, daily smoking, alcohol intake, depression symptoms, history of hypertension, history of type 2 diabetes, three or more daily servings of fruit intake, and two or more daily servings of vegetable intake. Bold values represent significant *p*-value < 0.05. PR—prevalence ratio; 95% CI—confidence interval at 95%.

## Data Availability

The data from the ENDES are publicly accessible on the INEI website: http://iinei.inei.gob.pe/microdatos/ (accessed on 10 February 2023).

## Appendix A

**Table A1:** Simple and adjusted multivariate regression analysis of factors associated with normal-weight abdominal obesity phenotype by sex.

Characteristics	Male		Female	
	Crude Model PR (CI 95%)	Adjusted Model * PR (CI 95%)	Crude Model PR (CI 95%)	Adjusted Model * PR (CI 95%)
**Age (years)**				
18 to 35	Ref.	Ref.	Ref.	Ref.
35 to 59	**4.46 (3.67–5.44)**	**4.64 (3.8–5.67)**	**1.57 (1.52–1.62)**	**1.56 (1.51–1.62)**
60 to 69	**7.32 (5.86–9.15)**	**7.77 (6.14–9.85)**	**1.60 (1.52–1.67)**	**1.58 (1.50–1.66)**
70 and older	**10.82 (8.81–13.27)**	**11.05 (8.83–13.84)**	**1.62 (1.55–1.70)**	**1.53 (1.45–1.62)**
**Survey year**				
2018	Ref.	Ref.	Ref.	Ref.
2019	**1.29 (1.09–1.53)**	1.16 (0.98–1.37)	**0.88 (0.83–0.92)**	**1.12 (1.07–1.18)**
2020	1.02 (0.83–1.25)	0.87 (0.71–1.07)	**0.86 (0.81–0.90)**	**1.11 (1.05–1.17)**
2021	**1.22 (1.02–1.46)**	1.13 (0.95–1.34)	**0.82 (0.78–0.86)**	1.04 (0.98–1.09)
**Natural region**				
Metropolitan Lima	Ref.	Ref.	Ref.	Ref.
Rest of the coast	0.88 (0.75–1.03)	0.9 (0.77–1.07)	1.00 (0.96–1.04)	1.04 (1.00–1.08)
Andean	**0.45 (0.37–0.53)**	**0.54 (0.44–0.67)**	**0.89 (0.85–0.92)**	0.96 (0.92–1.00)
Amazon	**0.47 (0.38–0.59)**	**0.66 (0.51–0.85)**	**0.95 (0.90–0.99)**	1.01 (0.96–1.06)
**Educational level**				
No education	Ref.	Ref.	Ref.	Ref.
Primary	2.11 (0.3–14.66)	2.25 (0.32–15.69)	1.18 (0.95–1.48)	1.18 (0.94–1.49)
Secondary	1.46 (0.21–10.12)	2.43 (0.35–16.98)	0.98 (0.78–1.22)	1.10 (0.87–1.38)
Higher	2.15 (0.31–14.93)	2.97 (0.42–20.79)	1.00 (0.8–1.25)	1.08 (0.86–1.36)
**Wealth index**				
Poorest	Ref.	Ref.	Ref.	Ref.
Poor	**1.51 (1.2–1.88)**	**1.94 (1.47–2.56)**	**1.17 (1.11–1.23)**	**1.20 (1.13–1.29)**
Middle	**1.88 (1.5–2.36)**	**2.43 (1.76–3.35)**	**1.10 (1.04–1.16)**	**1.09 (1.02–1.18)**
Rich	**2.47 (1.99–3.07)**	**2.53 (1.83–3.51)**	**1.21 (1.15–1.28)**	**1.18 (1.09–1.27)**
Richest	**3.50 (2.83–4.32)**	**3.03 (2.16–4.25)**	**1.26 (1.19–1.32)**	**1.18 (1.09–1.27)**
**Residence area**				
Urban	Ref.	Ref.	Ref.	Ref.
Rural	**0.59 (0.50–0.70)**	**1.49 (1.16–1.93)**	**0.87 (0.83–0.90)**	**0.93 (0.87–0.99)**
**Daily smoking**				
No	Ref.	Ref.	Ref.	Ref.
Yes	1.21 (0.84–1.75)	0.89 (0.62–1.28)	**1.18 (1.02–1.37)**	1.05 (0.91–1.21)
**Alcohol intake**				
No	Ref.	Ref.	Ref.	Ref.
Yes	1.12 (0.94–1.32)	**1.2 (1.01–1.41)**	0.94 (0.88–1.00)	0.97 (0.91–1.03)
**Depressive symptoms**				
No	Ref.	Ref.	Ref.	Ref.
Yes	**1.21 (1.03–1.42)**	1.08 (0.92–1.26)	0.96 (0.93–1.00)	**0.94 (0.91–0.97)**
**Hypertension**				
No	Ref.	Ref.	Ref.	Ref.
Yes	**3.08 (2.59–3.66)**	**1.21 (1.00–1.45)**	**1.31 (1.26–1.37)**	**1.07 (1.03–1.12)**
**Type 2 diabetes**				
No	Ref.	Ref.	Ref.	Ref.
Yes	**3.00 (2.37–3.80)**	1.05 (0.82–1.35)	**1.44 (1.37–1.50)**	**1.14 (1.08–1.19)**
**Fruit intake ≥3 serving/day**				
No	Ref.	Ref.	Ref.	Ref.
Yes	**0.51 (0.42–0.62)**	**0.63 (0.52–0.76)**	0.98 (0.94–1.02)	0.98 (0.95–1.02)
**Vegetable intake ≥2 serving/day**				
No	Ref.	Ref.	Ref.	Ref.
Yes	0.79 (0.60–1.04)	0.81 (0.63–1.05)	**1.11 (1.06–1.17)**	**1.09 (1.04–1.14)**

* Adjusted for age, survey year collection, natural region, educational level, wealth index, residence area, daily smoking, alcohol intake, depression symptoms, history of hypertension, history of type 2 diabetes, three or more daily servings of fruit intake, and two or more daily servings of vegetable intake. Bold values represent significant *p*-value < 0.05. PR—prevalence ratio; 95% CI—confidence interval at 95%.
